# High-Resolution Characterization of Protein-Conjugated, mRNA-Loaded Lipid Nanoparticles by Analytical Ultracentrifugation

**DOI:** 10.1002/adfm.202523042

**Published:** 2025-11-22

**Authors:** Sophia Bird, Connor Smith, Nahal Habibi, Samantha Rivera, Saeed Mortezazadeh, Reece Martin, Benjamin M. Geilich, Gilles Besin, Borries Demeler

**Affiliations:** Department of Chemistry and Biochemistry, University of Lethbridge, Lethbridge, AB T1K4M3, Canada; Orbital Therapeutics, Cambridge, MA02142, USA; Orbital Therapeutics, Cambridge, MA02142, USA; Orbital Therapeutics, Cambridge, MA02142, USA; Department of Chemistry and Biochemistry, University of Lethbridge, Lethbridge, AB T1K4M3, Canada; Department of Chemistry and Biochemistry, University of Lethbridge, Lethbridge, AB T1K4M3, Canada; Orbital Therapeutics, Cambridge, MA02142, USA; Orbital Therapeutics, Cambridge, MA02142, USA; Department of Chemistry and Biochemistry, University of Lethbridge, Lethbridge, AB T1K4M3, Canada; Department of Chemistry and Biochemistry, University of Montana, Missoula, MT59812, USA

**Keywords:** analytical ultracentrifugation, custom grid, lipid nanoparticle analysis, sedimentation velocity, UltraScan

## Abstract

The study describes a novel use for the Custom Grid (CG) algorithm in UltraScan targeting lipid nanoparticles (LNPs) with cargos ranging from empty LNPs, LNPs loaded with messenger RNA (mRNA), and LNPs conjugated with proteins, or both. The CG method is used to fit sedimentation velocity analytical ultracentrifugation experiments performed in density matching mode to derive partial specific volume, molar mass, and hydrodynamic radius distributions for LNPs. Because LNP cargos often differ in density from the encapsulating lipids, density (or partial specific volume) is a critical quality attribute to quantify LNP composition and cargo loading. It is shown that the CG approach, in combination with D_2_O density matching, faithfully fits even complex cases that exhibit both sedimenting and floating analytes in the same sample without sacrificing generality, and derives density distributions confirming successful cargo loading. In addition, the method provides distributions for hydrodynamic radii, molar mass, and sedimentation coefficients. Analysis of the same samples with the parametrically constrained spectrum analysis provides orthogonal validation in good agreement with the CG analysis. The results show that polydispersity assessment and other metrics alone are unreliable in determining the fraction of empty LNPs present in a formulation, but density profiles obtained here clearly distinguish mRNA-loaded from empty LNPs.

## 1. Introduction

Lipid Nanoparticles (LNPs) have emerged as a pivotal platform for the delivery of diverse therapeutic modalities, including small interfering RNA (siRNA),^[1,2]^ messenger RNA (mRNA),^[3]^ and small-molecule drugs such as doxorubicin (Doxil).^[4]^ Recent advances in lipid nanoparticle-based RNA delivery technologies are reshaping the therapeutic landscape, as exemplified by the clinical success of siRNA-based LNPs (Onpattro) and mRNA-based LNP vaccines (Spikevax and Comirnaty). These novel platforms have broad potential applications ranging from the development of vaccines for infectious diseases to therapies for rare genetic disorders, to personalized immunotherapies, and treatments for other complex diseases.^[5,6]^ LNPs are modular assemblies typically composed of ionizable lipids, helper lipids, cholesterol, and polyethylene glycol (PEG) lipids,^[7, 8]^ each contributing distinct structural and functional roles.^[1]^ Compositional modifications, including lipid chemistry, molar ratio adjustments, or incorporation of additional components such as permanently charged selective organ targeting lipids,^[9]^ have been shown to modulate biodistribution in vivo.^[1]^ In addition to modulation of lipid components, LNPs can be functionalized with targeting moieties, such as peptides,^[10]^ antibodies,^[11]^ or aptamers,^[12]^ on their surface to promote cell-type-specific delivery. However, this design space that enables extensive compositional and structural tuning, and thereby functional versatility, can also introduce structural and compositional heterogeneity.

The self-assembly of LNPs typically yields a heterogeneous population with respect to both particle size and cargo load, such that some LNPs are loaded with RNA and others are completely empty. As a result, not all LNPs contribute equally to the drug’s efficacy. In the case of mRNA, the cargo itself can be heterogeneous in composition, presenting heterogeneity in size due to degradation or premature termination of the transcription process, as well as conformational variability due to hybridization of single-stranded, complementary RNA regions on the same or another mRNA molecule. When hybridization between different mRNA molecules occurs, interlinking and aggregation of mRNA cargo are also possible.

A key design element in RNA-LNP development is the ratio of nitrogen in the ionizable lipid to phosphates in the nucleic acid (N/P), and there is an optimal range required to ensure complete complexation, payload protection, efficient cytosolic delivery, and low toxicity.^[13]^ At the optimal N/P, the LNP morphology exhibits a dense, uniform core with enhanced colloidal stability. A previously reported Multiwavelength Analytical Ultracentrifugation (MW-AUC) method exploited differences in density and spectral properties of lipids and nucleic acids to distinguish nucleic acid-loaded LNPs from empty LNPs, which was a critical gap in LNP characterization.^[14]^ These results also showed that siRNAs loaded into LNPs at two different N/P ratios could be resolved by density matching AUC. In another example, Li et al. demonstrated that a conventional Dlin-MC-based LNP formulation encapsulated, on average, 2 mRNA molecules per loaded LNP, while 40–80% of the population consisted of empty LNPs depending on the assembly and nanoprecipitation conditions, as documented by multi-laser cylindrical illumination confocal microscopy spectroscopy (CICS), which enabled single-particle resolution of cargo loading heterogeneity.^[15]^

LNP size is an important parameter influencing biodistribution and cellular uptake.^[16]^ Typical sizes range from 50 to 150 nanometers (nm), making them ideal for drug delivery applications and compatible with standard sterilizing-grade filtration during clinical manufacturing. Particles smaller than ≈100 nm can traverse liver sinusoidal fenestrae, whereas larger particles (>100 nm) may favor extrahepatic delivery by reducing hepatic accumulation.^[16]^ Li et al. also demonstrated that LNP size can impact mRNA payload capacity using CICS. When Dlin-MC-based LNPs were reduced in size by increasing their PEG content, fewer mRNA copies per LNP were detected.^[15]^

Properties such as encapsulation efficiency, size distribution, particle density, and subpopulation distribution (including the relative abundance of empty versus RNA-loaded LNPs and conjugated versus unconjugated LNPs) are critical determinants of potency, manufacturability, and stability. However, these properties can be challenging to measure directly on intact LNPs under native conditions. Standard characterization of RNA-loaded LNPs includes measurement of particle size and polydispersity index (PDI) by dynamic light scattering (DLS), with typical acceptance criteria requiring a hydrodynamic diameter below 150 nm and a PDI below 0.2. A variety of biophysical solution-based methods are currently in use to characterize LNP formulations, including DLS, cryo transmission electron microscopy (cryo-TEM), particle counters employing total internal reflection fluorescence (TIRF), size exclusion chromatography (SEC), and nanoparticle tracking analysis (NTA). Each method has its advantages, but none provide a comprehensive, high-resolution, first-principles characterization. Each method also demonstrates distinct drawbacks, which include the requirement for labeling and standardization (TIRF), the inability to separate molecules based on size or density (DLS, NTA), the acquisition of only a weight averaged value (DLS), the inability to separate by density (SEC, NTA), the lack of quantitative bulk observations the failure to identify relevant cargo loading (cryo-TEM),^[14]^ and the failure to provide the necessary resolution (DLS, SEC) to draw important conclusions about cargo load. Furthermore, unincorporated active pharmaceutical ingredients may not be detected or identified with sufficient resolution (DLS, cryo-TEM, TIRF), distorting the weight average metrics reported for LNPs. In addition, low-resolution metrics such as the PDI and average hydrodynamic radius obtained by DLS can fail to reliably describe important quality attributes relevant to the efficacy and safety of LNPs, which are necessary for a comprehensive characterization of the formulation.

This study illustrates that Analytical Ultracentrifugation (AUC) alone can comprehensively characterize all key solution metrics, removing the necessity for various characterization techniques, by establishing an AUC-based framework as a powerful platform for analyzing complex LNPs comprising both mRNA payloads and protein-functionalized surfaces. We demonstrate that AUC effectively resolves LNP subpopulations in native solutions with greater resolution than the combined output of multiple orthogonal techniques. This capability directly assesses cargo loading distributions, density distributions, and the differentiation between protein-conjugated and unconjugated LNP subpopulations. In addition, coupling sedimentation/density-matching AUC with fluorescence detection revealed that more than 80% of empty LNPs and more than 90% of mRNA-loaded LNPs were successfully decorated with protein, and further allowed us to distinguish between empty and RNA-loaded particles in a manner not achievable with conventional size-based metrics.

Collectively, these findings position AUC as a cutting-edge analytical tool based on first principles for the characterization of LNPs in academic and industrial environments. This advancement presents new opportunities to forge strong structure–function relationships in this rapidly evolving field.

## 2. Theory

### 2.1. Custom Grid Approach

**Figure 1.**
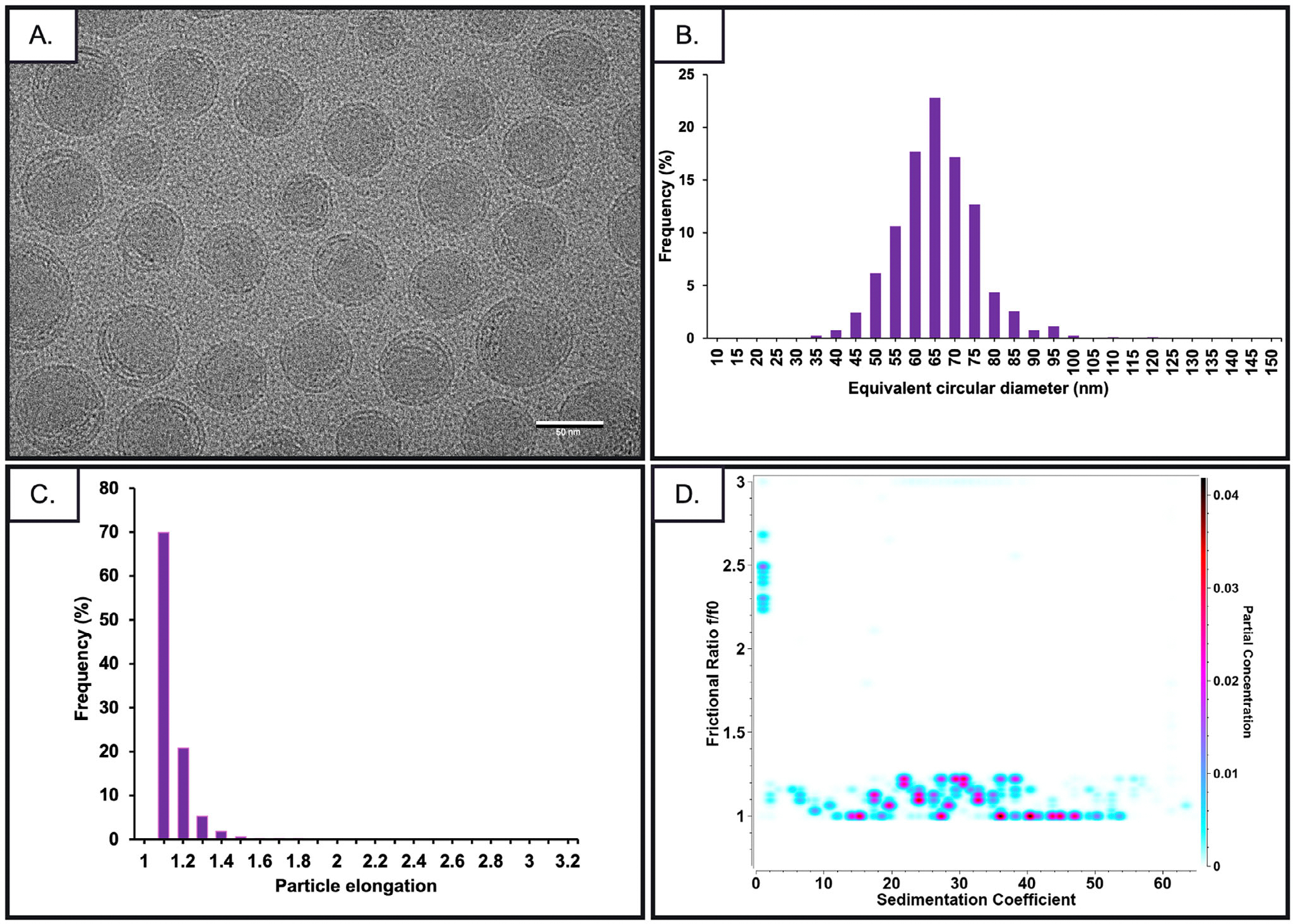
Morphological and size characterization of LNPs. A) Cryo-EM image of LNPs. B) Histogram of equivalent circular diameters for individual LNP particles. C) Distribution of particle sphericity, calculated as the ratio of major to minor axis lengths. D) Pseudo-3D plot of 2DSA-MC of LNP-P mRNA in 0% D_2_O.

To address characterization challenges, we previously introduced new methods based on density matching analytical ultracentrifugation (AUC)^[14]^ to simultaneously identify the heterogeneity in size and cargo load for RNA-LNPs. During density matching, the solvent density is gradually increased by adding D_2_O to the light water-based solvent to shift the sedimentation distributions of the analyte(s) toward the point of equi-buoyancy. In cases where the density of the analytes is only slightly higher than that of the solvent, the shift in solvent density causes the analytes to float. When mixtures of analytes with variable density are sedimented, conditions can arise where both sedimenting and floating species are observed. Here we describe the use of the Custom Grid (CG) module implemented in UltraScan (see Section S1, Supporting Information) to provide a generalized first-principle model for the variable buoyancy of LNPs whose density can be both higher and lower than the solvent density in the same sample, which current sedimentation velocity fitting methods, including those described in,^[14]^ cannot correctly accommodate, because they assume constant partial specific volume. Samples containing molecules that are both higher and lower in density than the solvent’s density, simultaneously float and sediment, corresponding to both positive and negative sedimentation coefficients. Datasets including significant contributions from both sedimenting and floating species had to be excluded from the analysis in,^[14]^ requiring adjustment of D_2_O concentration to make samples either entirely sedimenting or floating. The sign of the sedimentation transport is controlled by the buoyancy term, $1-\overset{‾}{v}\rho$, which is positive when the product of the solvent density, ρ, and the partial specific volume, $\overset{‾}{\nu }$, is less than 1.0. Sedimentation occurs when the density of the sedimenting particle is higher than the density of the solvent. Flotation (negative sedimentation) occurs when this product exceeds 1.0. In such a case, the density of the sedimenting particle is lower than the density of the solvent, turning the buoyancy term negative. When the buoyancy term equals zero, the density of the analyte is matched by the solvent density, and no sedimentation transportis observed, regardless of molar mass, and only diffusion transport occurs. Traditional sedimentation velocity modeling approaches assume a constant $\overset{‾}{\nu }$ and rely on grid-based parameterizations of the size and shape domain to derive linear combinations of sedimentation and diffusion coefficient pairs representing the sedimenting sample. Optimization relies on finite element solutions of the Lamm equation^[17,18]^ using a constant partial specific volume for all species. In the general case, however, only the density of the solvent remains constant, not the partial specific volume of the particle distribution. Used initially to parameterize sedimentation experiments of metal nanoparticles,^[19]^ the CG approach has been adapted here to span the positive and negative buoyancy terms and accommodate samples that contain both sedimenting and floating samples. This is achieved by combining multiple grids covering regions with variable partial specific volume. In addition, the parameter range can be further constrained based on prior knowledge, for example, based on the frictional ratio of particles observed in cryo-TEM experiments. Importantly, assumption of a constant shape factor, which is well supported by the cryo-TEM results, ensures that our approach remains entirely general, and does not require invalid assumptions such as a constant weight-average diffusion coefficients as is done in Sedfit,^[20]^ which is untenable when samples exhibit particle diameter distributions ranging from 20 to 110 nm as the empty particles examined in this study (see Figure 1B). Furthermore, the approach chosen in Sedfit prevents the derivation of reliable hydrodynamic radii and molar mass distributions, because they depend on the knowledge of accurate diffusion coefficient distributions (see Equations (4) and (7) below).

In AUC experiments, sedimentation and diffusion transport are modeled with the Lamm^[21]^ Equation (1):

(1) $${\left(\frac{\partial C}{\partial t}\right)}_{r}=\frac{-1}{r}\frac{\partial }{\partial r}{\left[s\omega^{2}r^{2}C-Dr\frac{\partial C}{\partial r}\right]}_{t}$$

where C is the observed concentration of a solute in the AUC cell as a function of time t and radius r, subject to boundary conditions of t>0 and m<r<b where m is the meniscus of the solution column, and b is the bottom of the AUC cell. The sedimentation and diffusion coefficients are denoted by s and D, respectively, and ω denotes the angular velocity. The concentration of each solute in a mixture is determined by fitting a 2D linear combination of n Lamm equation solutions $L_{i}$ spanning all expected s and D values to the experimental data using a non-negatively constrained least squares fitting approach^[22]^ which determines the amplitude $c_{i}$ of each s-D pair in the linear combination Equation (2):

(2) $$C_{T}=\sum_{i}^{n}c_{i}L\left[S_{i},D_{i}\right]$$

The sedimentation coefficient, s, is expressed by the Svedberg equation (Equation (3)):

(3) $$s=\frac{M(1\;-\;\overset{‾}{v}\rho )}{Nf}$$

where M denotes the molar mass, N denotes Avogadro’s number, and f represents the frictional coefficient of the solute. We define the partial specific volume, $\overset{‾}{v}$, as the apparent partial specific volume of a sedimenting particle observed in solution, including hydration and any ions bound, divided by the sum of all density increments of analyte, solvent, and ions bound. The diffusion coefficient is expressed by Equation (4):

(4) $$D=\frac{RT}{Nf}$$

where R is the universal gas constant and T is the absolute temperature. Because the temperature is accurately measured in an AUC experiment, the diffusion coefficient directly yields the frictional coefficient of the solute. Combining Equation (3) with Equation (4) also yields the molar mass, provided the partial specific volume of the solute and the density of the solvent are known Equation (5):

(5) $$M=\frac{sRT}{D(1\;-\;\overset{‾}{v}\rho )}$$

The molar mass, together with the partial specific volume, also provides the volume of a single sedimenting particle Equation (6):

(6) $$V=\frac{M\overset{‾}{v}}{N}$$

The Stokes-Einstein Equation (7) relates the frictional coefficient of the solute to the Stokes radius (also called hydrodynamic radius, $R_{h}$) of the solute:

(7) $$R_{h}=\frac{f}{6\pi \eta }$$

where η is the viscosity of the solvent.

Setting V from Equation (6) equal to the volume of a sphere, the hypothetical minimal radius of the solute, $R_{0}$, can be derived, and used to calculate the hypothetical minimal frictional coefficient, $f_{0}$, of this sphere using the Stokes–Einstein Equation (8):

(8) $$f_{0}=6\pi \eta {\left(\frac{3V}{4\pi }\right)}^{1/3}$$

It is important to note that in our interpretation, the volume V relates to the volume of the entire sedimenting particle, including hydration and any ions bound. For our purposes, this allows us to more accurately compare the frictional coefficient of the entire sedimenting particle (which is observed in our measurements) to the hypothetical minimal sphere, which then corresponds to the same volume. In other interpretations, only the volume of the anhydrous analyte is considered. Comparing the actual frictional coefficient, f, to the frictional coefficient, $f_{0}$, of the hypothetical minimal sphere, we define the frictional ratio α of the solute Equation (9) to be:

(9) $$\alpha =\frac{f}{f_{0}}$$

This limits α to a theoretical minimal value of unity, reflecting the shape isotropy of a perfect sphere, and introduces a slight bias on the molar mass by including the hydration of the sedimenting particle. Given the large heterogeneity in the molar mass distributions in our LNP measurements, this minor bias does not present any concern in the accuracy of our molar mass distributions. When heterogeneity is large, the signal for the diffusion coefficient from each individual species becomes rather small, decreasing the reliability of D measurements. To address this challenge, Equations (2–9) can now be used to re-parameterize s and D in terms of the frictional ratio, frictional coefficient, molar mass, and partial specific volume. However, these parameterizations are not possible unless additional knowledge is available. For example, in order to determine the molar mass, the partial specific volume of each solute must be known (see Equation (5)). The same is true for the frictional ratio, which can only be determined if the partial specific volume is known. If the fitting grid is to be constructed using the sedimentation coefficient and the frictional ratio α, a parameterization of D is expressed as a function that depends on the viscosity and density of the solvent, as well as s,α, and $\overset{‾}{v}$:

(10) $$D=RT{\left[N18\pi (\alpha \eta {)}^{3/2}{\left(\frac{s\overset{‾}{v}}{2(1\;-\;\overset{‾}{v}\rho )}\right)}^{1/2}\right]}^{-1}$$

To fit experimental data, a range for s and D is necessary, and must be specified by the analyst. While the s range is readily estimated from model-independent methods like the enhanced van Holde–Weischet method,^[23]^ or the time derivative method,^[24]^ a reasonable range for D is often not easily estimated. Traditionally, the D range is specified by using a parameterization of D employing a constant $\overset{‾}{v}$ value and a variable frictional ratio, α, which can be estimated based on the approximate anisotropy range of the particle (see Equation (10)). For LNPs, cryo-TEM results often confirm a nearly isotropic shape for all particles in the mixture, allowing us to parameterize D by fitting a variable $\overset{‾}{v}$ range, using a fixed anisotropy instead. Viscosity and density for many aqueous buffers are readily available through programs like UltraScan^[25]^ or Sednterp,^[26]^ which also correct them for the effects of run temperatures not at standard conditions (20 °C). By considering the latter adjustment, it is now possible to treat the partial specific volume as a variable parameter, and fitting it instead of D or α. By making this change, we can approach the general case of a variable partial specific volume without introducing user bias into the fitting range. Similarly, rearranging Equation (5) and solving for D, it is also possible to parameterize D using the molar mass M, which requires that $\overset{‾}{v}$ remains constant.

### 2.2. Software Implementation

The Custom Grid (CG) approach is available in UltraScan and allows users to build 2D basis grids for s and D, replacing s or D with a matrix of alternative combinations (see Section S2, Supporting Information). The software further allows the user to combine two or more subgrids, which may be based on different combinations, into a combined grid that can be solved using the 2D spectrum analysis.^[27,28]^ Combined grids can be saved by the software into the UltraScan LIMS database, and processed by the UltraScan Science Gateway on large-scale high-performance computing infrastructures.^[29]^ To support efficient parallelization, the user can visually select suitable subgrid configurations to solve the optimization problem on parallel computers, generating coarser subgrids for each CPU that together span the entire range of the combined grid, achieving complete coverage of the search space, while at the same time ensuring that the grid does not exhaust the available memory on the computer. Employing an iterative approach, the amplitudes in the linear combination of all grid points in all subgrids are successively refined to produce a high-resolution result, covering the entire search space at the desired granularity. By specifying multiple grids with positive and negative buoyancy configurations, the user can now cover systems that not only vary in terms of particle density or partial specific volume, but also control fitting parameters that extend across positive and negative sedimentation ranges to accommodate mixtures with heterogeneous distributions of LNPs that sediment and float simultaneously. Such a grid is shown in Section S3 (Supporting Information). For very heterogeneous solutions, a logarithmic grid spacing is also supported, for example, to accommodate molar mass distributions ranging over multiple orders of magnitude. This supports modeling of very large aggregates or fast sedimenting LNPs that exhibit little to no diffusion. In such cases, a high-resolution diffusion grid wastes compute cycles and unnecessarily slows down the fitting process. A low resolution or single-dimensional s-value grid is sufficient to achieve good fits, especially for heterogeneous systems often encountered in LNP preparations.

## 3. Results

### 3.1. Hydrodynamic and Morphological Characterization of Protein-Conjugated RNA-LNP

As shown by cryo-TEM imaging and ImageJ analysis (Figure 1A–D), the frequency distribution of LNP equivalent circular diameters indicates that most particles fall within the 45–85 nm range (Figure 1B), with the highest frequency around 65 nm, reflecting a relatively narrow and symmetric size distribution. Further analysis of LNP shape based on particle elongation, calculated as the ratio of the major axis to the minor axis, showed that most particles exhibited elongation values between 1.0 and 1.2 (Figure 1C), consistent with a predominantly spherical morphology. This is an orthogonal confirmation of LNP sphericity, providing helpful constraints for AUC-based modeling. In agreement with the cryo-TEM results, sedimentation velocity analysis by 2DSA-MC also indicated that LNP-P-mRNA samples have a frictional ratio close to 1.0, indicating isotropy, and showed an evenly distributed pattern of frictional ratio signal ranging between 1.0 and 1.2 (Figure 1D). For subsequent analysis, the frictional ratio was fixed to 1.1 to constrain the diffusion coefficients for the 2DSA analysis using Custom Grid models.

### 3.2. Fluorescence AUC

**Figure 2.**
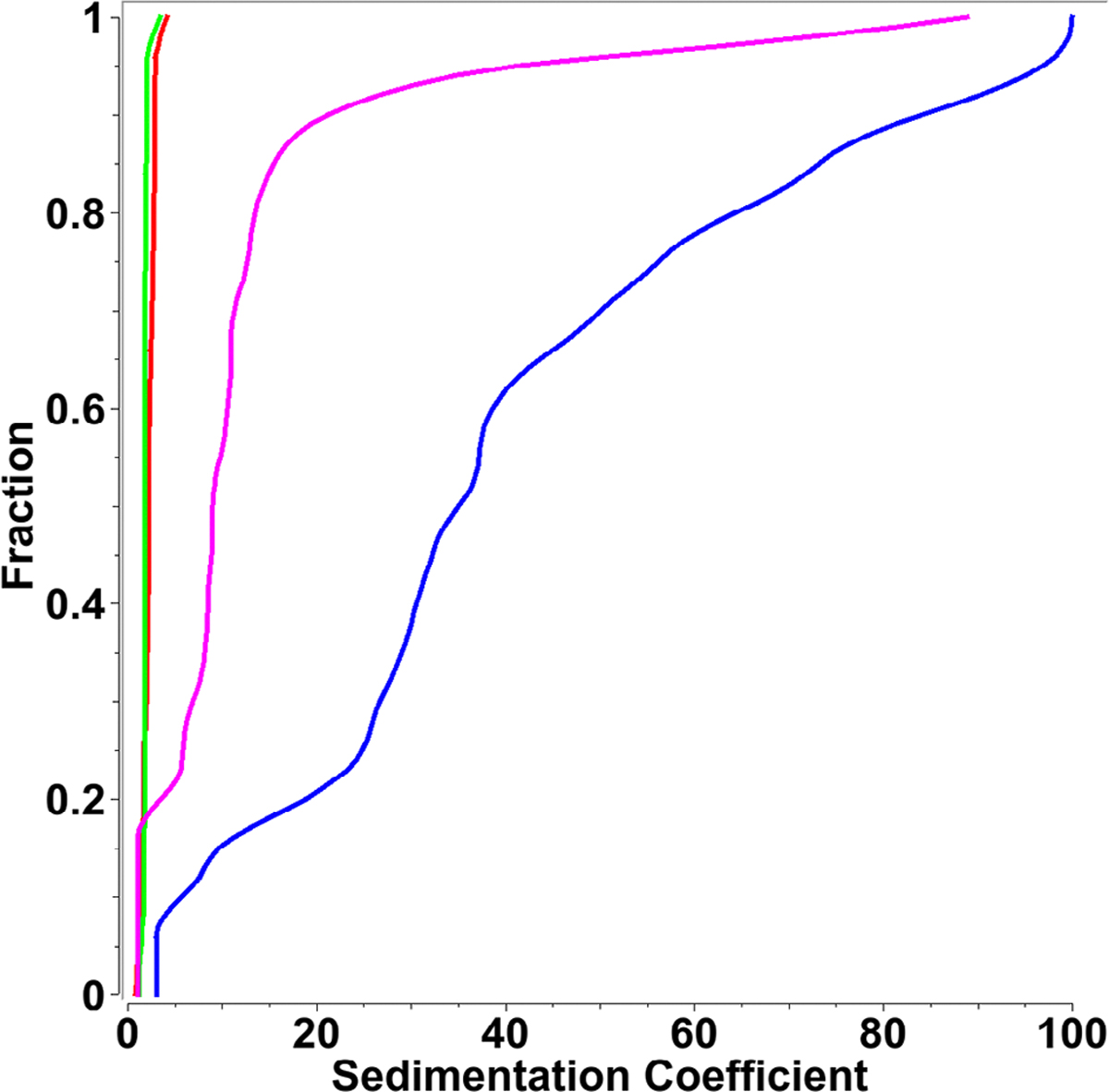
Integration of protein into empty LNPs. Green: unlabeled protein measured at 280 nm with absorbance optics. Red: Fluorescently labeled protein measured at 280 nm with absorbance optics. Magenta: LNPs complexed with fluorescently labeled protein. Blue: LNPs complexed with fluorescently labeled protein as well as mRNA. Magenta and Blue were measured with Aviv fluorescence detection.

To measure the protein’s conjugation efficiency to the LNP’s surface, the protein was fluorescently labeled and tracked using the Aviv fluorescence optics retrofitted into a Beckman Proteomelab XLA. To ensure that the fluorescent label does not alter the solution behavior of the protein itself, the unlabeled and labeled proteins were measured by themselves using UV absorbance detection at 280 nm. LNPs conjugated with the fluorescently labeled proteins were then measured in light water by fluorescence detection. Since LNPs by themselves are not fluorescent, only LNPs successfully conjugated with the fluorescently labeled protein can be detected in the fluorescence optical system. In addition, any unincorporated protein free in solution will also be visible. We tested both empty LNPs conjugated with fluorescently labeled protein, as well as LNPs loaded with mRNA cargo and conjugated with fluorescently labeled protein (see Figure 2), which showed <20% of free protein in the LNP-P sample, and <10% free protein in the LNP-P-mRNA sample based on co-sedimentation rates with the control.

### 3.3. Fitting of Sedimentation Velocity Data

During density matching, we encountered buffer conditions where the sample contained molecules both denser and less dense than the buffer, causing some molecules to sediment and others to float in the same sample. The Custom Grid approach was used to model these conditions with a grid that spanned partial specific volume ranges consistent with floating and sedimenting molecules. A representative example for such a buffer condition can be found in an LNP sample loaded with mRNA at 15% D_2_O (Figure 4). Fits based on the identical custom grid for higher and lower D_2_O concentrations for mRNA loaded LNPs that are purely floating or sedimenting are shown in Figures S4–S6 (Supporting Information). Excellent fits were obtained, resulting in very low RMSD values of 0.00212 absorbance units at 300 nm and random residuals, corresponding to a signal-to-noise ratio of ≈450. Here, the boundary shapes at low radius values reflect sedimenting species, while at the bottom of the cell at higher radii, the boundaries reflect floating species. Boundary positions in the middle of the cell are at the isopycnic point, where no sedimentation transport occurs. Hence, these boundary positions have a horizontal (baseline) appearance. Over time, their concentration decreases, as sedimentation and flotation transport push material to the meniscus and bottom position of the cell. Similar results were obtained for purely sedimenting and floating samples, all using the same Custom Grid.

### 3.4. Density Matching

**Figure 3.**
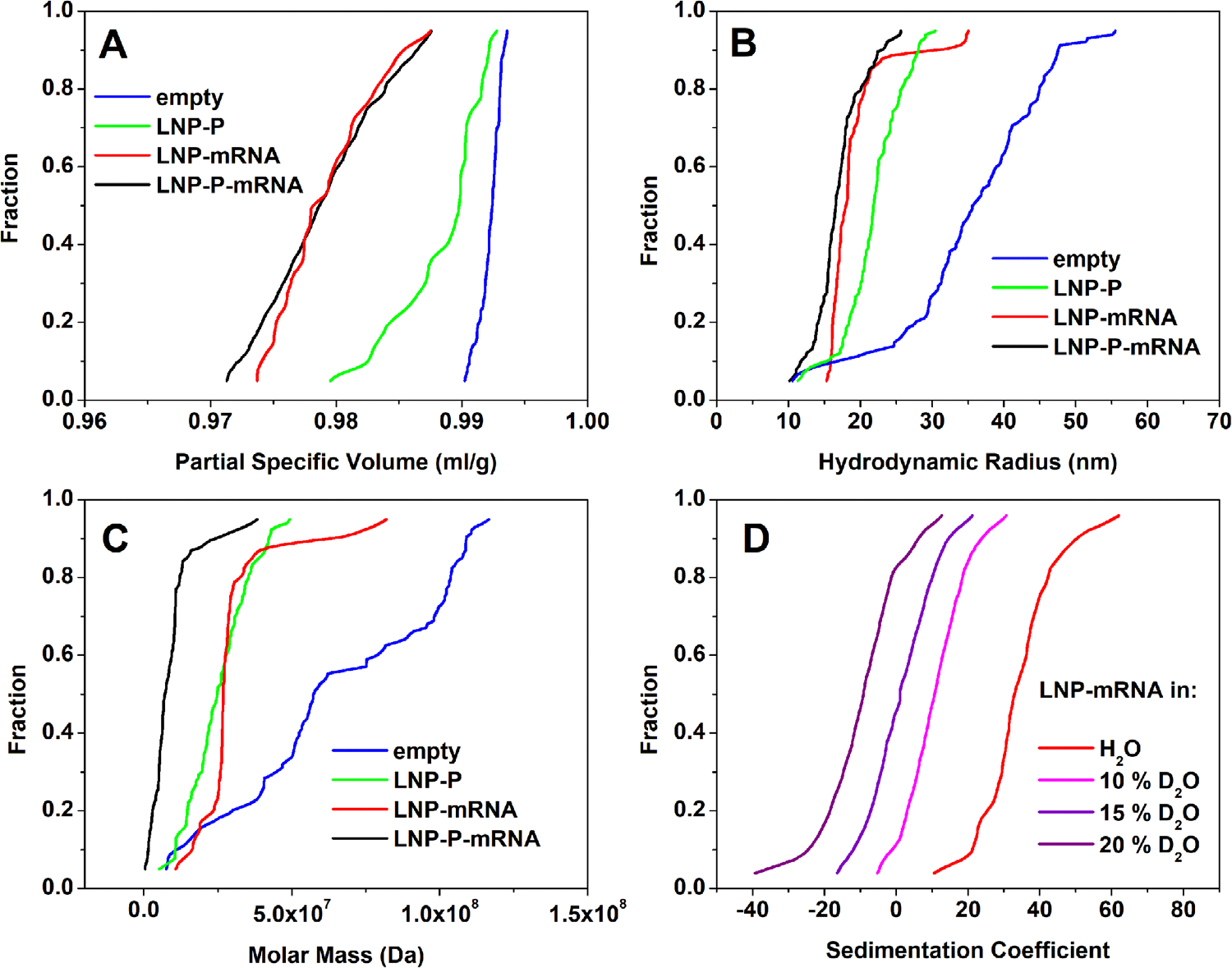
Hydrodynamic characterization of lipid nanoparticles (LNPs). A) Partial specific volume distributions of empty LNPs (blue), LNPs conjugated with protein P (green), LNPs loaded with mRNA cargo (red) and LNPs loaded with mRNA as well as conjugated to protein P (black). B) Hydrodynamic radius distributions for LNPs in (A). C) Molar mass distributions for LNPs in (A). D) Representative sedimentation coefficient distributions for LNPs loaded with mRNA as a function of D_2_O concentration, demonstrating the left shift due to increasing density when the D_2_O concentration is increased Red: in light water (0 % D_2_O). Pink: in 10 % D_2_O. Violet: in 15% D_2_O. Purple: in 20 % D_2_O.

raditional AUC experiments report on the sedimentation and diffusion coefficients of the measured analytes. This alone is not sufficient to uniquely identify mass, shape, and density of analytes; a third parameter is required to obtain additional information. This information is gleaned from density matching experiments, which utilize measurements in multiple buffer densities to derive a partial specific volume distribution for all analytes in the mixture, providing the third variable to uniquely solve Equations (3),(5),(6),(8–10). The universal applicability of the same Custom Grid for both sedimenting and floating samples, and those that exhibit simultaneous floating and sedimenting transport, makes the Custom Grid approach ideal for density matching experiments of lipid nanoparticles, where the sedimentation patterns change from sedimenting LNPs in light water to floating LNPs in higher D_2_O concentrations. Therefore, we were able to fit all density matching experiments with the same grid and apply consistent metrics to the fitting approach for all samples. The primary observable from the density matching experiment is a partial specific volume distribution of the sample, which can be used to derive the molar mass distribution of the sample according to Equation (5). Sedimentation and diffusion coefficients measured in the sedimentation velocity experiment serve to predict the hydrodynamic radius, $R_{h}$, (Equation (7)). A frictional ratio value of 1.1 was used to constrain the diffusion coefficient according to Equation (10) as a function of $\overset{‾}{v}$ and s, which produced very good fits. The partial specific volume distributions of empty LNPs, empty LNPs conjugated with protein, mRNA loaded LNPs, and mRNA loaded LNPs conjugated with protein are shown in Figure 3A–D, the hydrodynamic radius distributions are shown in Figure 3B, and the molar mass distributions are shown in Figure 3C. Sedimentation coefficient distributions for LNPs loaded with mRNA as a function of D_2_O concentrations are shown in Figure 3D.

### 3.5. Comparison of Custom Grid Results with PCSA

The parametrically constrained spectrum analysis (PCSA) aims to constrain the finite element models to univalued, 2D functional relationships.^[30]^ Such functions can frequently describe the appropriate hydrodynamic parameter space for heterogeneous systems. For example, aggregating proteins like amyloid-*β* polymerize end-to-end to form elongated fibrils, or mixtures of different length DNA molecules produce specific size-shape dependencies that can be expressed in parameterized functions for heterogeneous mixtures that follow these well defined relationships. We hypothesized that a straight-line parameterization for the partial specific volume as a function of sedimentation coefficient can be applied to fits of AUC data from LNP formulations. The PCSA fits were compared to the results obtained from the Custom Grid approach for mRNA loaded LNPs in PBS containing 15% D_2_O, shown in Figure 5. Compared to an excellent RMSD of 0.00212 for the Custom Grid analysis, the PCSA analysis achieved an acceptable RMSD value of 0.00393 with the same data. While sedimentation distributions were nearly identical (see Figure 5A), slight discrepancies between the straight-line constrained PCSA models and the unconstrained custom grid models were apparent in 2D plots for the partial specific volume domain (see Figure 5B). However, most of the signal aligned very well between the models.

## 4. Discussion

**Figure 4.**
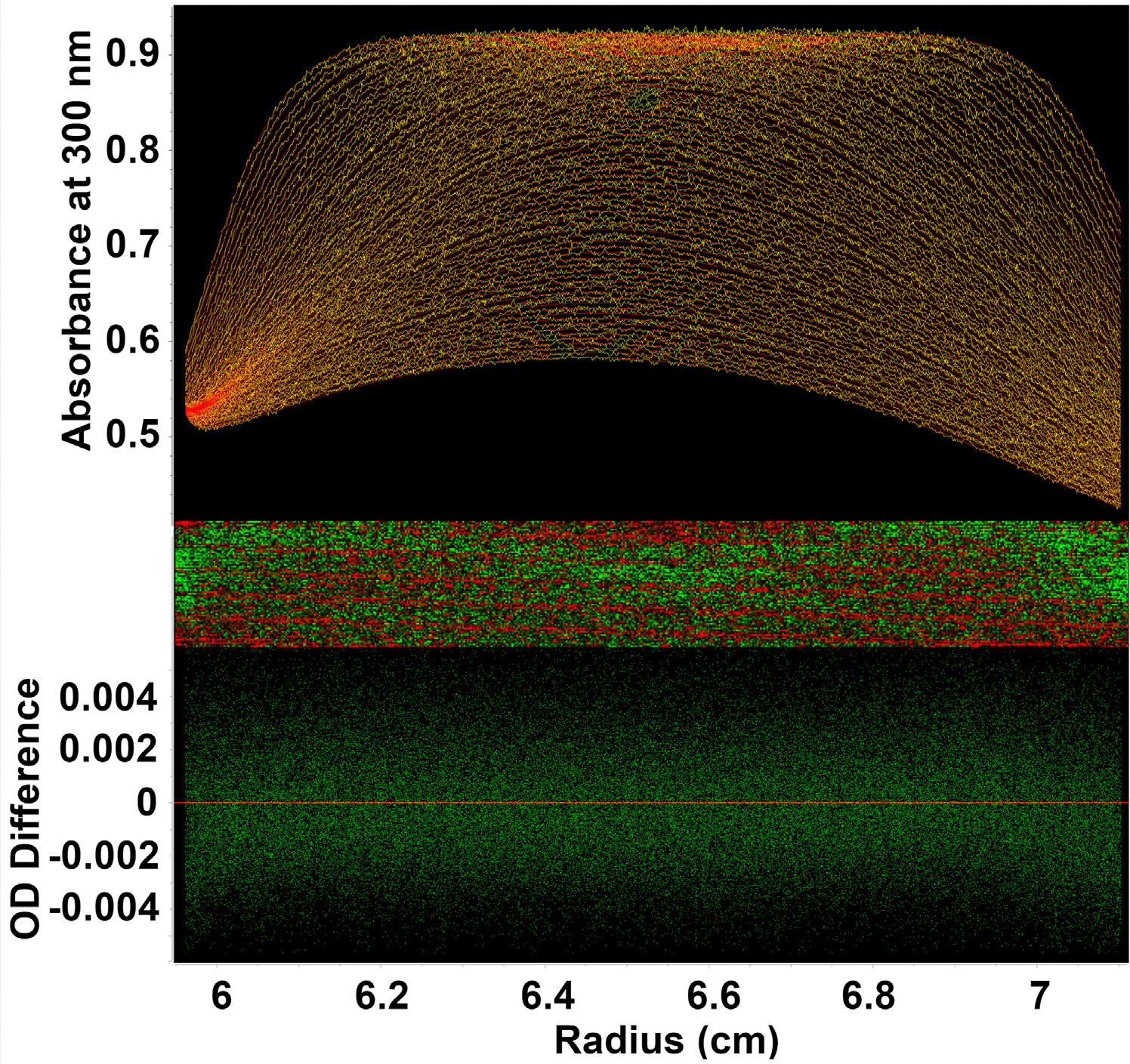
Sedimentation profile for mRNA loaded LNPs in 15% D_2_O. Top panel: Overlay of experimental sedimentation velocity data with a fitted 2DSAMonte Carlo model for LNP-mRNA, performed in PBS buffer using 15 % D_2_O. The 2DSA-MC analysis employed a Custom Grid model (see Methods), faithfully reproducing both sedimenting and floating particles present in the same sample. RMSD=0.00212 OD at 300 nm. Center panel: Residuals bitmap for this fit, displaying a random, grainy pattern without a recognizable pattern mismatch, indicating an excellent fit. Bottom panel: Residual plot of the above fit, demonstrating a random distribution without recognizable runs. Sedimentation profile overlays for mRNA loaded LNPs in buffer containing 0%, 10% and 20% D_2_O are shown in Supplemental Sections SI4-6.

**Figure 5.**
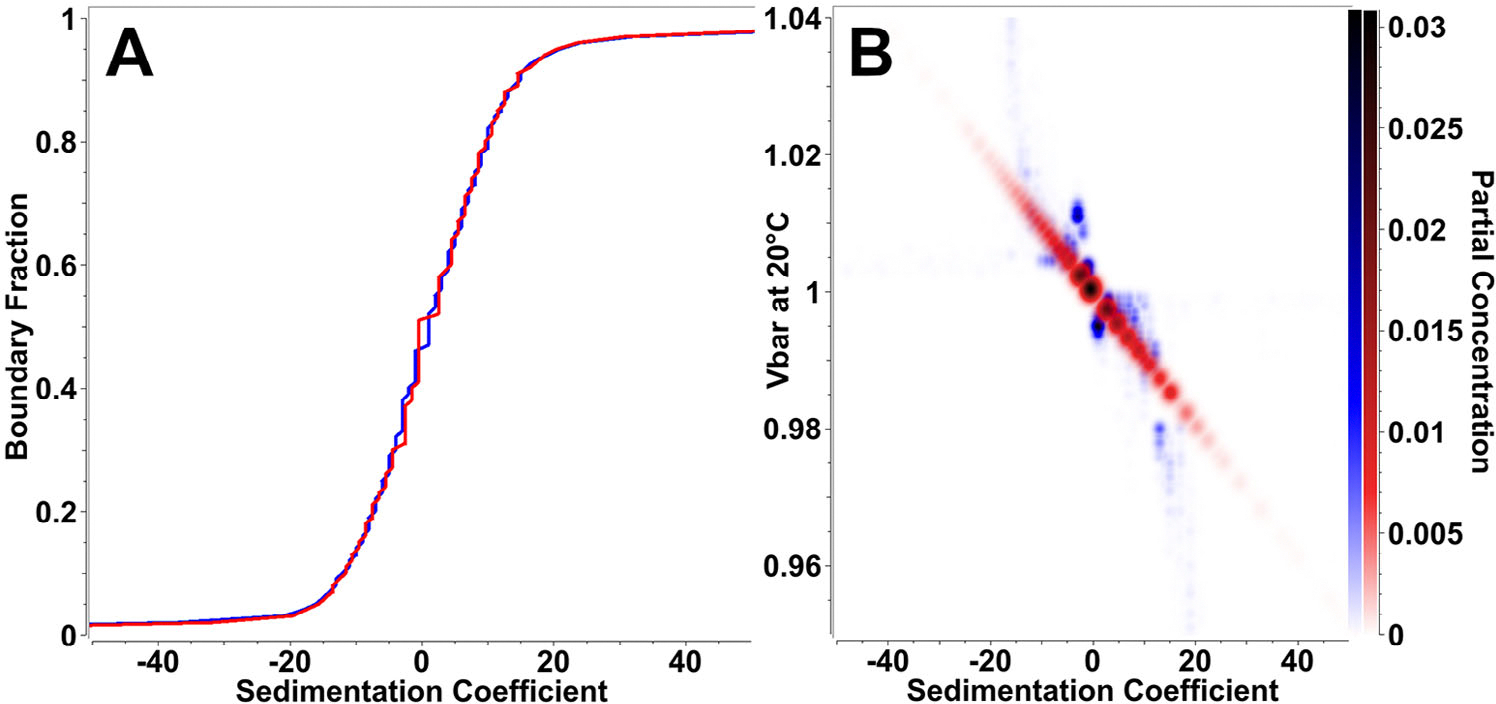
Comparison between the Custom Grid and the straight-line PCSA parameterization analysis. A) Sedimentation coefficient distributions for LNP-mRNA in 15% D_2_O, shown is an overlay between the two methods (red: PCSA, blue: Custom Grid). The distributions match almost exactly. B) 2D pseudo-3D representation of the partial specific volume distribution for LNP-mRNA in 15% D_2_O, shown is an overlay of the two methods (red: PCSA, blue: Custom Grid). The distributions show a high degree of similarity; however, the unconstrained Custom Grid approach detects a slight sigmoidal distribution of partial specific volume versus sedimentation coefficient, and achieves an improved RMSD value. Color density signifies partial concentration.

With the recent focus on RNA-LNP medicines like COVID-19 vaccines and cancer treatments, an increasing demand is placed on the characterization of such drugs. Existing methods can fail to fully distinguish critical quality attributes such as cargo loading and particle size distribution. Cargo load often differs in density from the encapsulating lipid shell, making particle density an essential parameter for quantifying cargo loading within LNPs. The prevalence of LNP-based therapeutics demands a standardized approach for quality control of LNP characterization that provides a comprehensive evaluation of all variable parameters of LNPs, such as hydrodynamic radius, density (=cargo load), and molar mass, mainly when Good Manufacturing Practice (GMP) standards must be observed to ensure the safety and efficacy of the drug. Combined with density matching and constraints confirmed through cryo-TEM analysis (see Figure 1A), the Custom Grid approach presented here simultaneously provides high-resolution quantification of all three parameters (see Figure 3A–C). The first-principle nature of analytical ultracentrifugation further eliminates the need for any reference standards, and the results generated from AUC experiments are absolute, provided the instrument functions correctly. The same custom grid could be used successfully to fit four different LNP formulations, suspended in four different buffer solutions, proving the generality of this fitting approach. Furthermore, excellent residuals were obtained by this method, which exceeded the PCSA method, which, unlike other methods commonly used, can fit a 2D hydrodynamic space. While the PCSA and Custom Grid analysis produced similar results (see Figure 5), the Custom Grid analysis provided superior fitting quality (see Figure 4). We propose that the PCSA method should be extended to provide additional parameterizations to accommodate the sigmoidal nature of the partial specific volume distribution shown in Figure 5B for the Custom Grid analysis, which is currently not fully captured by the PCSA, leading to measurable differences in the RMSD. Nevertheless, the Custom Grid analysis resulted in excellent agreement with the PCSA analysis (Figure 5), and led to the same conclusions, suggesting that even a properly parameterized PCSA is a worthwhile alternative for fitting AUC experiments of LNP formulations.

### 4.1. LNP Characterization

The three primary metrics for LNP characterization are the partial specific volume, the hydrodynamic radius, and the molar mass distribution. We compared four cargo loading states (empty, LNP-P, LNP-mRNA, and LNP-P-mRNA) using density matching experiments fitted with the Custom Grid analysis. Our results show that the partial specific volume distribution of empty LNPs provided a mostly homogeneous partial specific volume distribution with the lowest density between 0.990–0.992 mL g^−1^. LNPs conjugated to a protein (LNP-P) were slightly more heterogeneous in their density distribution, and also denser than the empty LNPs (0.980–0.990 mL g^−1^). LNPs loaded with mRNA (LNP-mRNA) were significantly denser than empty or LNP-P formulations, and slightly more heterogeneous in density (0.974–0.985 mL g^−1^). When protein conjugated LNPs were loaded with mRNA (LNP-P-mRNA), the overall density and heterogeneity in density did not change appreciably from the LNP-mRNA formulation(0.972–0.985 mLg^−1^). These results are summarized in Figure 3A. In contrast, the hydrodynamic radius distribution for the empty LNPs suggested a large, heterogeneous range of particle sizes, from 20–110 nm in diameter. Surprisingly, the heterogeneity and the size were reduced significantly once the LNPs were conjugated with protein (30–50 nm diameter) or loaded with mRNA (30–60 nm diameter), or both (20–40 nm diameter), suggesting a reorganization of the LNP structure once conjugated with protein and/or loaded with cargo. The LNP-P-mRNA formulation appeared to be the smallest and had the least heterogeneity in size. This pattern was closely matched by the molar mass distribution, which was also most heterogeneous for the empty LNP formulation (7.5 × 10^6^ – 1.2 × 10^8^ Da), followed by the LNP-mRNA formulation (1.1 × 10^7^ – 8.2 × 10^7^ Da), LNP-P (5.0 × 10^6^ – 4.9 × 10^7^ Da), and the LNP-P-mRNA formulation (3.0 × 10^5^ – 3.8 × 10^7^ Da). Fluorescence AUC demonstrated high protein conjugation efficiency of >80% for LNP-P and >90% for LNP-P-mRNA samples.

These results indicate that relying solely on size for polydispersity index measurements does not fully capture the heterogeneity of LNP formulations. For example, both hydrodynamic radius and molar mass measurements show significant overlap between empty LNPs and mRNA-loaded LNPs, suggesting an empty LNP population within the mRNA-loaded formulations. However, when partial specific volume is considered, it is clear that there is no overlap between the empty and mRNA-loaded LNPs, demonstrating that there are no particles in the mRNA-loaded formulations that are purely empty. This observation mirrors our earlier measurements on siRNA-loaded LNPs.^[14]^ Therefore, the most comprehensive and reliable assessment of LNP heterogeneity requires the measurement of partial specific volume distributions using AUC density matching approaches, coupled with Custom Grid analysis.

## 5. Experimental Section

### Lipid Nanoparticle Formulation and Surface Modification:

Orbital Therapeutics produced lipid nanoparticles (LNPs) and messenger RNA (mRNA). Briefly, LNPs were formulated by dissolving lipid components in an ethanolic phase and mixing them with an aqueous solution at a pH below the pKa of the ionizable lipid, using a 1:3 ethanol-to-aqueous volume ratio. mRNA was synthesized by in vitro transcription and purified by FPLC chromatography. For RNA-loaded LNPs (LNP mRNA), the aqueous phase contained mRNA at the desired concentration, while for empty LNPs, the same buffer was used without mRNA. Rapid mixing was carried out in a controlled mixing chamber, followed by buffer exchange via dialysis to reach the final formulation buffer. Protein was conjugated to the LNPs using a chemical conjugation strategy (LNP-P mRNA). For mRNA-loaded LNPs, conjugation stoichiometry was calculated based on mRNA concentration. For empty LNPs, the LNP content was matched to mRNA-loaded formulations by adjusting the sample to achieve a similar absorbance at 450 nm, and conjugation stoichiometry was applied accordingly (LNP-P). Unreacted components were removed by size exclusion chromatography. Final LNP samples were concentrated via centrifugation to a target concentration and used for subsequent analyses and characterization. To enable fluorescence-based detection of surface-conjugated protein on LNPs, the protein of interest was directly labeled using a DyLight 488 NHS ester according to the manufacturer’s instructions prior to conjugation.

### Physicochemical and Functional Characterization of LNPs:

LNP size and PDI were measured by DLS using a Dynapro Plate Reader iii (Wyatt Technology) and software provided by the manufacturer. The intensity-weighted hydrodynamic diameters and PDIs were recorded for each formulation to assess size distribution and sample polydispersity. Encapsulation efficiency of mRNA within LNPs was determined using the QuantiT RiboGreen RNA assay. Samples were incubated in the presence or absence of 2% Triton X-100 to distinguish between total and unencapsulated RNA. Following incubation, RiboGreen reagent was added, and fluorescence was measured using a spectrophotometer (Ex/Em = 480/520 nm). To calculate encapsulation efficiency, the fluorescence signal from the untreated samples (representing unencapsulated RNA) was subtracted from the signal of Triton X-100-treated samples (representing total RNA), divided by the total RNA signal, and multiplied by 100.

For LNP cryo-TEM, Quantifoil holey carbon grids (R1.2/1.3, 400 mesh, Cu) were glow-discharged at 20 mA for 20 s. A 4 μL aliquot of LNP sample was applied to each grid and flash-frozen in a liquid ethane/propane mixture following manual blotting for 3–4 s. Grids were imaged using a Talos Arctica (FEI, Hillsboro, OR) transmission electron microscope operated at 200 keV, equipped with a K3 Summit detector (Gatan, Warrendale, PA) and a BioContinuum imaging filter. Images were recorded at 39 000× magnification (2.249 Å/pixel) with a defocus range of −3 μm. Particle morphology was analyzed manually in ImageJ^[31]^ across 5–7 images, yielding a total of 780 particles. Size and shape distributions were quantified based on equivalent circular diameter and particle elongation metrics to assess uniformity and spherical morphology.

### Analytical Ultracentrifugation:

All analytical ultracentrifugation (AUC) experiments were conducted at the Canadian Center for Hydrodynamics at the University of Lethbridge (Lethbridge, Alberta). UV-intensity data was collected in a Beckman Coulter Optima AUC, and fluorescence intensity data was collected using a Beckman Proteomelab XLA retrofitted with a fluorescence detector using 488 nm excitation (Aviv Biomedical). Fluorescent samples were loaded into 3 mm titanium centerpieces fitted into fluorescence cell housings (Nanolytics, Potsdam, Germany). All experiments were run at 30 krpm for 10 hours at 20 °C using an An50Ti rotor. Density matching experiments with empty LNPs, LNP-P, LNP mRNA, and LNP-P mRNA were conducted to determine partial specific volume ($\overset{‾}{v}$) distributions. A 1 mL aliquot of H_2_O 20 x Phosphate Buffered Saline (PBS) was evaporated in a vacuum chamber and reconstituted with 1 mL of D_2_O. Each LNP sample was prepared at identical concentration in H_2_O/D_2_O mixtures containing 0%, 10%, 15%, and 20% D_2_O, adjusting the D_2_O-based PBS concentration to 1x before mixing with pure D_2_O. Empty LNPs were measured at ≈0.58 OD at 280 nm, while the LNP-P, LNP-mRNA, and LNP mRNA samples were measured at ≈0.83 OD at 300 nm. Samples with fluorescently labeled protein (A488) were measured by fluorescence AUC to test if the protein was conjugated to the LNPs or free in solution. A488 LNP-P and A488 LNP-P mRNA samples were measured using fluorescence optics at a concentration of 0.6 OD at 295 nm. An unlabeled and A488 labeled protein control were measured using UV-intensity optics at 0.5 OD and 0.2 OD at 280 nm, respectively. All concentrations are reported for a 1 cm pathlength. Sedimentation velocity data were fitted using the 2D spectrum analysis (2DSA)^[27,28]^ using the Custom Grid (2DSA-CG) method. For all samples, a grid covering sedimentation coefficients ranging from −100 to −1 s, and from +1 to +100 s. The partial specific volume was varied from 1.003 to 1.05 mL g^−1^ for the floating portion of the grid, and from 0.94 to 0.999 mL g^−1^ for the sedimenting portion of the grid, holding the frictional ratio constant at 1.1. The grid resolution was set to 200 grid points for the s-value range, and 120 points for the partial specific volume range, for a total of 24 000 grid points to be fitted, using 82 subgrids for the parallelization.^[27]^ Sedimentation velocity data were fitted in a multi-step refinement process to remove timeand radially-invariant noise, and to determine the meniscus and bottom positions of the solution column. A further refinement step uses the iterative 2DSA-CG-IT (IT) analysis to optimize time- and radially invariant noise at the fitted boundary conditions.^[32]^ To evaluate the impact of experimental noise on the obtained 2DSA-CG-IT distributions, a 100 iteration Monte Carlo analysis was performed in combination with the Custom Grid approach as the last fitting step.^[33,34]^ The Monte Carlo models were also used for the density matching analysis and to derive hydrodynamic radius distributions.^[35]^ Density matching experiments were performed as described in.^[14]^ Briefly, to obtain $\overset{‾}{\nu }$ distributions, sedimentation distributions from Monte Carlo models from each H_2_O:D_2_O ratio were integrated and extrapolated against the solution density for each boundary position to zero sedimentation, identifying the point where the density of the analyte equals the density of the solvent. For these extrapolations, the sedimentation coefficients were corrected for buffer viscosity, but not for solvent density at each H_2_O:D_2_O ratio.^[14]^ Parametrically constrained spectrum analysis (PCSA) was used to assess frictional ratio and $\overset{‾}{\nu }$ variations.^[30]^ When fitting the $\overset{‾}{v}$ value, the frictional ratio was fixed at 1.1, constraining the anisotropy to mostly spherical particles for consistency with cryo-TEM imaging results.

## Supplementary Material

Supplemental Information

Supporting Information is available from the Wiley Online Library or from the author.

## Data Availability

The data that support the findings of this study are openly available in Zenodo at https://doi.org/10.5281/zenodo.17009858, reference number [17009858].
