# Supplementary material for: High-Resolution Characterization of Protein-Conjugated, mRNA-Loaded Lipid Nanoparticles by Analytical Ultracentrifugation
Source: Adv Funct Mater. Author manuscript; Available in PMC 2026 Jan 22. (PMC12823152; doi:10.1002/adfm.202523042)
Supplement: Supplemental Information [file NIHMS2130229-supplement-Supplemental_Information.pdf]

ADVANCED FUNCTIONAL MATERIALS

Supporting Information

for *Adv. Funct. Mater.*, DOI 10.1002/adfm.202523042

High-Resolution Characterization of Protein-Conjugated, mRNA-Loaded Lipid Nanoparticles by Analytical Ultracentrifugation

*Sophia Bird, Connor Smith, Nahal Habibi, Samantha Rivera, Saeed Mortezazadeh, Reece Martin, Benjamin M. Geilich, Gilles Besin and Borries Demeler**

Supplemental Information:

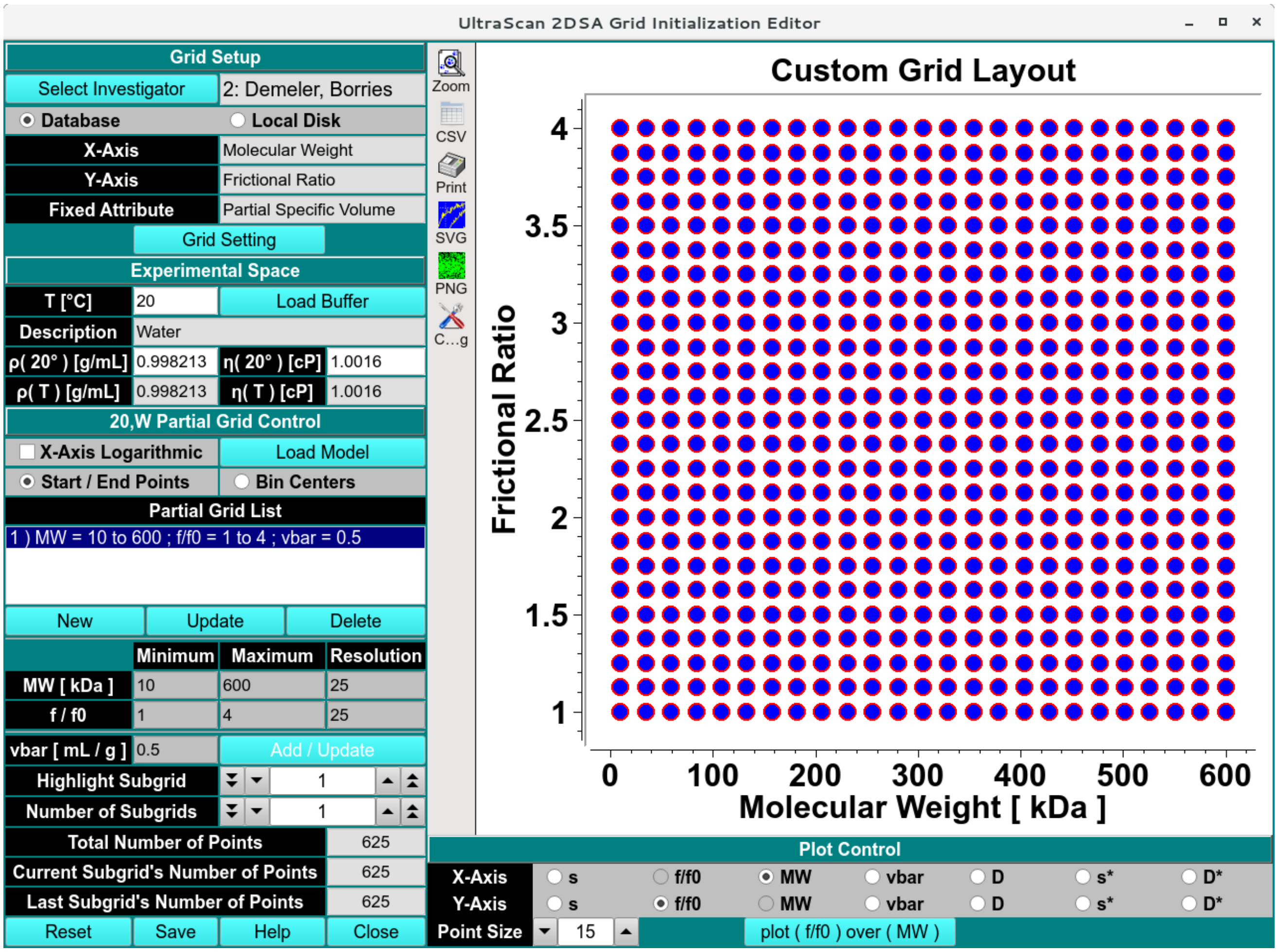

SI 1: Custom grid editor module in UltraScan. Controls allow defining a Custom Grid parameterization based on s, D, f/f0, molar mass and partial specific volume. Each Custom Grid requires two variable parameters and one fixed parameter. Different grids can be combined into a single grid, which can be used by the computational back end to fit experimental data. Internal logic prevents overlapping grids. A logarithmic distribution for each parameter is available, a selected grid can be displayed by changing one or both of the parameters to a different 2-dimensional representation, for example, a s vs. D plot with a fixed partial specific volume could be represented as a frictional ratio vs. molecular weight distribution. Existing Custom Grids can be loaded by the module and edited as needed and saved as a new model.

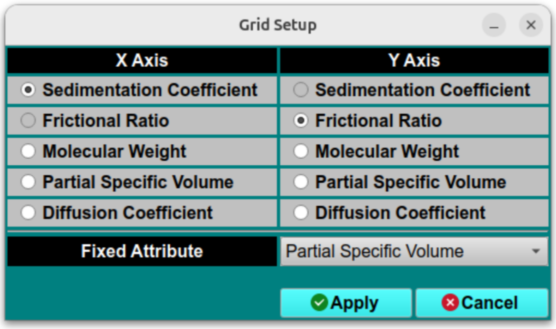

SI 2: Hydrodynamic parameterization of a two-dimensional fitting grid for s and D in the Custom Grid method, using alternative parameters Molecular Weight, Partial Specific Volume, and the frictional ratio. Every combination selected by the user requires a fixed parameter that must be held constant.

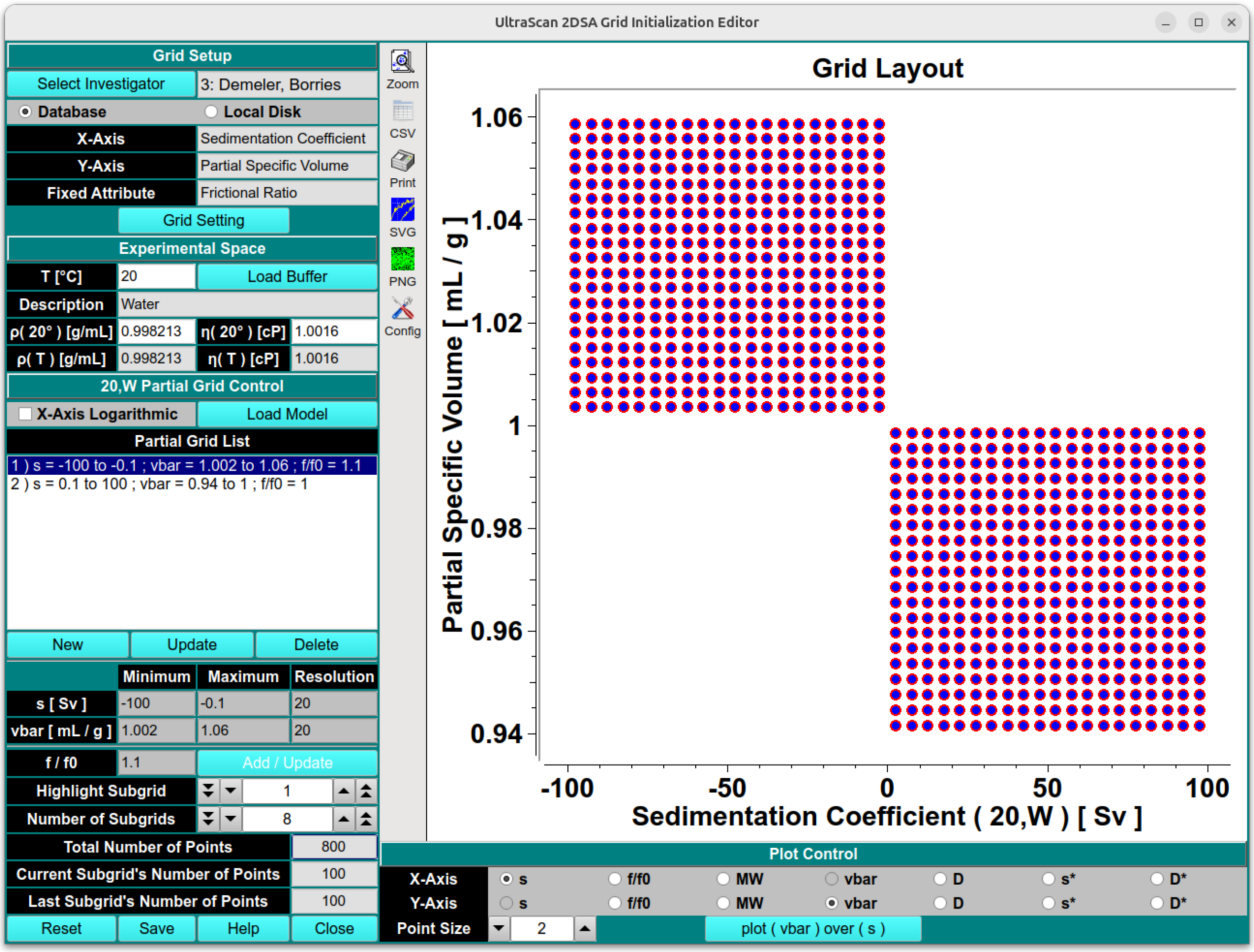

SI 3: Custom Grid layout for a typical LNP analysis for samples that contain both sedimenting (sedimentation is positive) and floating samples (sedimentation is negative). The resolution in these grids (20x20) is lower than recommended for a typical analysis for illustrative purposes, which should use 60-100 grid points for each grid, and each dimension.

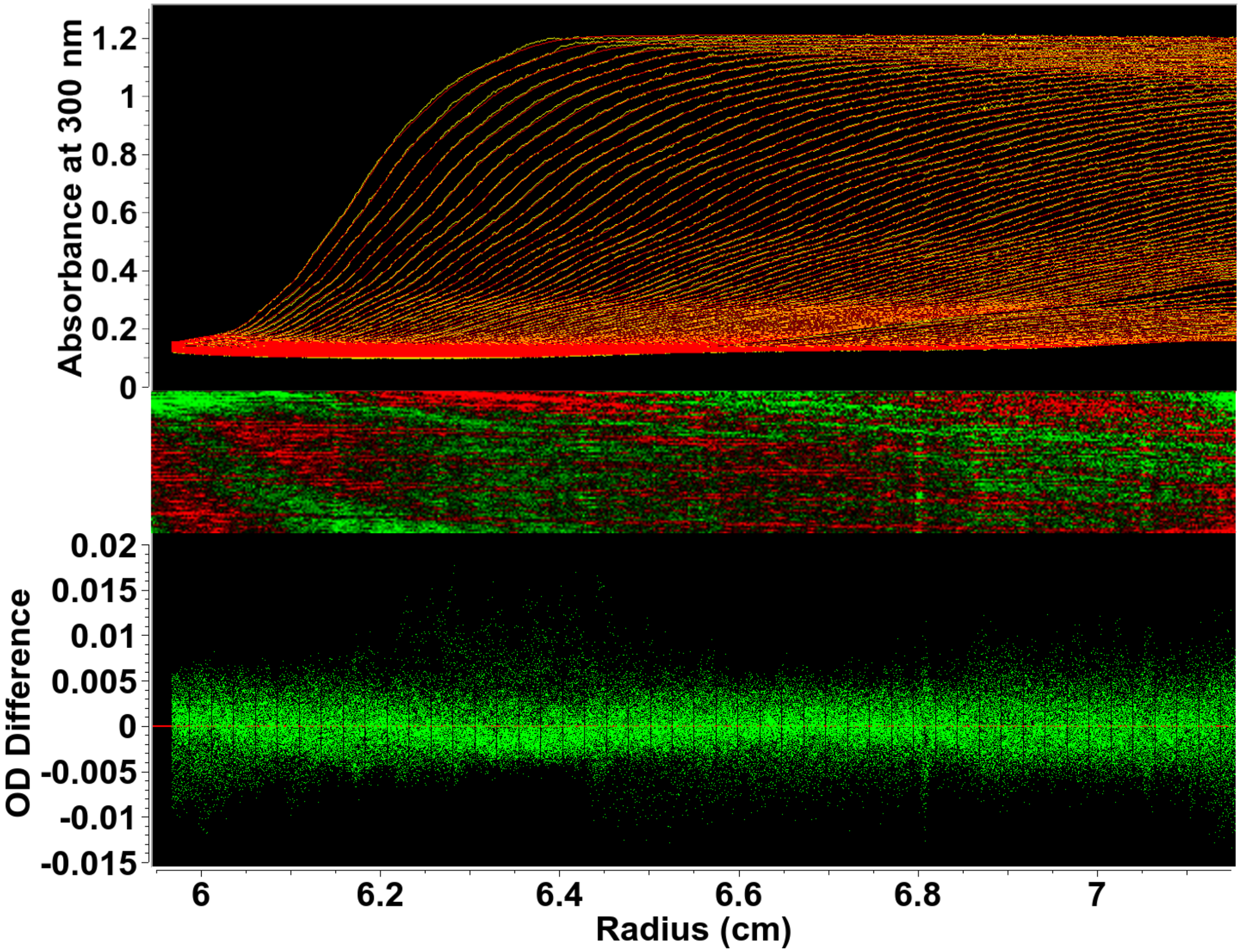

SI 4: Custom Grid analysis of the 0% D_2O sample for the mRNA loaded LNP showing only sedimentation

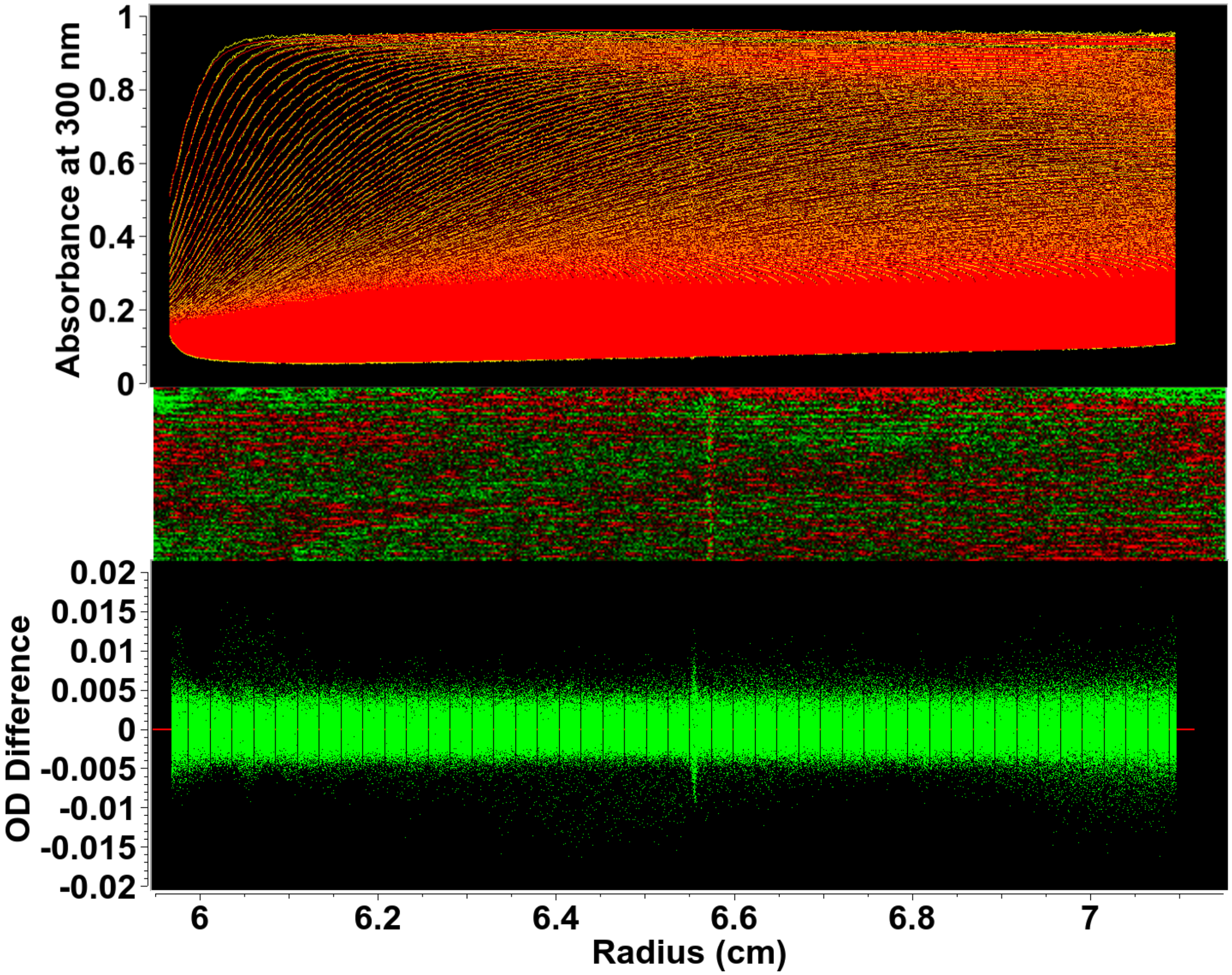

SI 5: Custom Grid analysis of the 10% D_2O sample for the mRNA loaded LNP showing mostly sedimentation and a small amount of floating material.

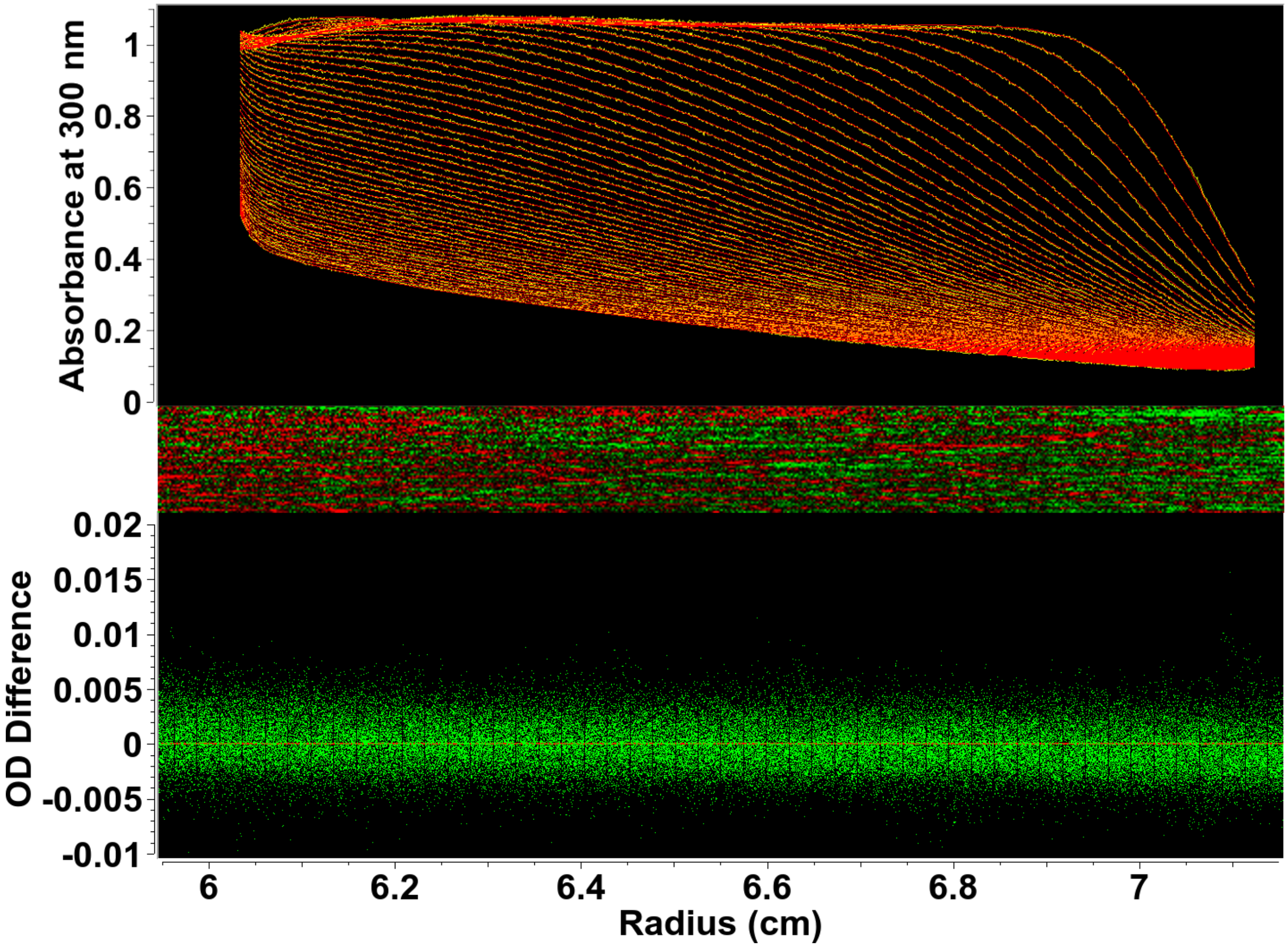

SI 6: Custom Grid analysis of the 20% D_2O sample for the mRNA loaded LNP showing only floating material.
